# White matter resection and verbal memory deficits after temporal lobe epilepsy surgery

**DOI:** 10.1093/braincomms/fcag033

**Published:** 2026-02-02

**Authors:** Lawrence P Binding, Davide Giampiccolo, Yaqi Ji, Marine Fleury, Sherry Liu, Lorenzo Bianchi, Anna Miserocchi, Andrew W McEvoy, Sallie Baxendale, Matthias Koepp, Fenglai Xiao, Aidan G O’Keeffe, Meneka K Sidhu, Peter N Taylor, Jane de Tisi, Gavin P Winston, John S Duncan, Sjoerd B Vos

**Affiliations:** UCL Hawkes Institute, Department of Computer Science, UCL, London, UK; Department of Epilepsy, UCL Queen Square Institute of Neurology, London, UK; Department of Epilepsy, UCL Queen Square Institute of Neurology, London, UK; Victor Horsley Department of Neurosurgery, National Hospital for Neurology and Neurosurgery, London, UK; Department of Psychology, University of California, Berkeley 94704, USA; Department of Epilepsy, UCL Queen Square Institute of Neurology, London, UK; Department of Epilepsy, UCL Queen Square Institute of Neurology, London, UK; Department of Neurosurgery, National Neuroscience Institute, Singapore 308433, Singapore; Medicine and Surgery, University of Bologna, Bologna 40125, Italy; Department of Epilepsy, UCL Queen Square Institute of Neurology, London, UK; Victor Horsley Department of Neurosurgery, National Hospital for Neurology and Neurosurgery, London, UK; Department of Epilepsy, UCL Queen Square Institute of Neurology, London, UK; Victor Horsley Department of Neurosurgery, National Hospital for Neurology and Neurosurgery, London, UK; Department of Epilepsy, UCL Queen Square Institute of Neurology, London, UK; Department of Epilepsy, UCL Queen Square Institute of Neurology, London, UK; MRI Unit, Chalfont Centre for Epilepsy, Chalfont St Peter, Greater London, UK; Department of Epilepsy, UCL Queen Square Institute of Neurology, London, UK; School of Mathematical Sciences, University of Nottingham, Nottingham, UK; Institute of Epidemiology and Health Care, UCL, London, UK; Department of Epilepsy, UCL Queen Square Institute of Neurology, London, UK; Department of Epilepsy, UCL Queen Square Institute of Neurology, London, UK; CNNP lab, School of Computing Science, Newcastle University, Newcastle NE4 5TG, UK; MRI Unit, Chalfont Centre for Epilepsy, Chalfont St Peter, Greater London, UK; Department of Epilepsy, UCL Queen Square Institute of Neurology, London, UK; MRI Unit, Chalfont Centre for Epilepsy, Chalfont St Peter, Greater London, UK; Department of Medicine, Division of Neurology, Queens University, Kingston, Canada, K7L 3N6; Department of Epilepsy, UCL Queen Square Institute of Neurology, London, UK; MRI Unit, Chalfont Centre for Epilepsy, Chalfont St Peter, Greater London, UK; UCL Hawkes Institute, Department of Computer Science, UCL, London, UK; Neuroradiological Academic Unit, UCL Queen Square Institute of Neurology, University College London, London WC1N 3BG, UK; Western Australia National Imaging Facility, The University of Western Australia, Nedlands 6009, Australia

**Keywords:** epilepsy, white matter, memory, epilepsy surgery

## Abstract

Temporal lobe resection for focal, drug-resistant temporal lobe epilepsy (TLE) causes verbal memory deficits in 30% of left hemisphere-operated patients. Structural, functional and computational modelling have shown a widespread structural and functional memory network with hubs in critical brain regions including the hippocampus, subcortical and neocortical regions. We hypothesized that damage to white matter pathways forming a network involving cortical and subcortical regions may be responsible for postoperative memory problems. In this study, we measured verbal memory encoding (immediate recall) and retrieval (delayed recall) outcome at three timepoints (preoperative, 3- and 12-month postoperatively) in 146 left TLE patients who underwent temporal lobe surgery and evaluated the impact of white matter tract section on verbal memory. Outcome was measured by the change in scores from preoperative to 3- and 12-month postoperatively and via the reliable change index. Utilizing resection masks from pre- and postoperative T1 scans, an atlas-based analysis utilizing reconstructions of the ventral cingulum and fornix confirmed these tracts involvement in verbal encoding but not retrieval. Using preoperative diffusion MRI (dMRI) reconstructions with resection masks to estimate the percentage of fibre bundle transection, we found that the ventral cingulum was significantly related to verbal encoding change and the fornix was related to verbal retrieval across both 3- and 12-month timepoints. Investigating volumes of ventral cingulum and fornix from postoperative dMRI reconstruction revealed that greater volume remaining of the ventral cingulum and fornix was related to less decline in verbal encoding but not retrieval. Our results suggest that verbal encoding may be supported by direct and indirect connections between the medial temporal lobe and subcortical regions with memory deficits arising from their transection. Verbal retrieval may rely on a greater neocortical network. These findings may inform a revised surgical approach to minimize damage to the fornix and ventral cingulum to optimize memory outcome, but recognizing the potential for worse seizure outcome with less ventral cingulum resections.

## Teaser

Temporal lobe surgery for epilepsy can cause verbal memory deficits. Binding *et al*. found that damage to the fornix and ventral cingulum, key white matter tracts in the memory network, is a significant factor. Cutting the ventral cingulum mainly impacts memory encoding, while cutting the fornix affects memory retrieval, suggesting different roles.

## Graphical Abstract

**Graphical Abstract:**
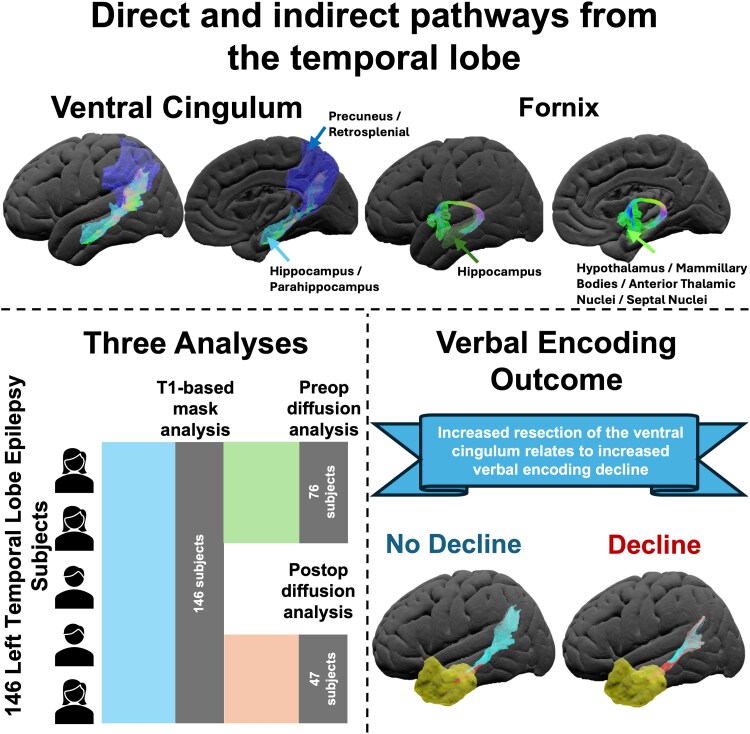

## Introduction

Temporal lobe resection is the treatment of choice for drug-resistant temporal lobe epilepsy (TLE).^1^ However, resection can result in a significant memory deficit in up to 30% of patients,^2-4^—particularly a verbal memory deficit after left temporal lobe resections.^3-6^ Typically, the hippocampus, amygdala and the anterior temporal lobe are resected during the surgery.^7^

Memory decline following temporal lobe resection has historically been linked to the hippocampal formation.^8^ However, procedures that spare the amygdala and hippocampus but lesion the parahippocampal gyrus have also resulted in severe memory impairment.^9^ This is also supported by functional MRI (fMRI) research suggesting that preservation of the parahippocampal gyrus may drive memory recovery postsurgically.^10,11^ The anterior thalamic nuclei have also been demonstrated to be vital for memory function.^12-16^ This is supported by research proposing a three-way interaction between the medial temporal lobe, medial diencephalic region and cortex.^15^ In combination, these findings suggest that the memory network is distributed.

**Figure 1:**
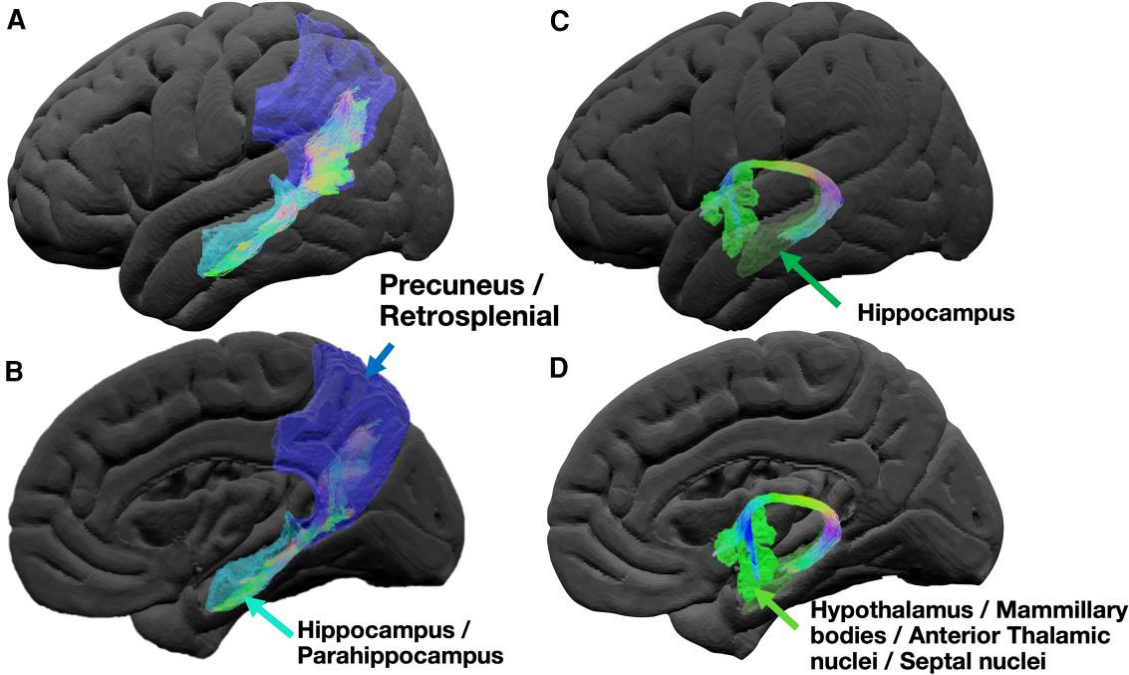
**Visualization of the ventral cingulum and fornix as viewed from lateral (A and C) and medial (B and D) viewpoints**. The ventral cingulum (**A** and **B**) connects the precuneus and retrosplenial (posterior cingulate) cortex with the hippocampus (dark green) and parahippocampus. The fornix (**C** and **D**) connects the hippocampus with the hypothalamus, mammillary bodies, anterior thalamic nuclei and septal nuclei.

White matter connections play a vital role in network function for transferring information. Diffusion MRI (dMRI) research suggests that white matter lesions, disrupting the wider network, can cause lasting deficits.^17^ The parahippocampal gyrus, hippocampus and anterior thalamic nuclei are all interconnected. The fornix subserves as a direct connection between the hippocampus to the anterior thalamic nuclei.^18^ The ventral cingulum, which connects the parahippocampal gyrus to the retrosplenial (posterior cingulate) cortex, provides an alternative projection from the medial temporal lobe to the anterior thalamic nucleus.^19^ These connections (Fig. 1) have been described as being the bridges which facilitate memory.^15^

Prior to surgery, the memory network in TLE is already functionally and structurally altered.^20,21^ The surgical intervention then acts as a second, powerful ‘insult’ that triggers further large-scale reorganization of the brain’s network in an attempt to maintain function.^22^ A recent review by Sainburg *et al*.^22^ comprehensively highlighted the widespread functional and structural changes that occur in the brain’s networks following resective epilepsy surgery. These changes are evidence of the brain's reorganization in response to the surgical lesion. For instance, the contralateral fornix can exhibit increased fractional anisotropy (FA; a measure of white matter integrity) post-surgery suggesting compensatory changes in the remaining network.^23^ The effects of surgery are not confined to the resected area. For example, despite not being resected, the anterior thalamic nuclei can undergo postoperative atrophy,^24^ highlighting how disconnecting a region's white matter tracts can disrupt the function of the wider memory network. This is further supported by findings that orbitofrontal functional connectivity is directly related to fornix tract FA, despite them not being connected.^21^ While these network changes are significant, there has been little investigation into the specific contribution of these white matter connections and their contribution to memory decline following resection in TLE.

Here, we investigate white matter pathways of 146 patients who underwent resection for left TLE and carry out extensive white matter analyses to determine whether these pathways are associated with preoperative memory and if damage to these pathways is associated with memory outcome. Understanding how damage to the cortico-subcortical memory network relates to memory and seizure outcome may inform revision of surgical approaches to increase memory preservation without adversely affecting seizure outcome.

## Material and methods

### Participants

We included MRI data from 146 patients who underwent left temporal lobe resection for focal, drug-resistant TLE at the National Hospital for Neurology and Neurosurgery, London, UK, between 1995 and 2019. Inclusion criteria were: no history of previous neurosurgery and a postoperative T1-weighted structural MRI (obtained between 3- and 12-month postoperatively). This project was approved by the Health Research Authority and London—Bloomsbury Research Ethics Committee (REC reference: 20/LO/0149; CAG number: 20/CAG/0013). All patients had the opportunity to opt out of research. This project did not carry any risk to participants and was conducted retrospectively on clinically acquired data.

This group of patients was split into three analysis groups depending on data availability: (i) tractwise voxel and atlas-based analysis cohort (*n* = 146) who had a postoperative structural 3D T1 MRI available, (ii) preoperative tract resection cohort (*n* = 71) who had a pre- and postoperative 3D T1 MRI as well as preoperative dMRI data and (iii) postoperative volumetric comparison cohort (*n* = 47) who had a pre- and postoperative 3D T1 and dMRI.

### Neuropsychology

All patients underwent standardized cognitive assessments of memory function. Before 2008, the Adult Memory and Information Processing Battery (AMIPB) was used, and after 2008, the BIRT Memory and Information Processing Battery (BMIPB) was utilized. Both consisted of an encoding and retrieval verbal recall task in which the patient is read a list of 15 common words, some of which are semantically related, and asked to recall as many as possible. The total number of words recalled over five trials is recorded (max 75) for the encoding task. The patient is then presented with a second distractor list of 15 words and asked to recall as many as possible. Following the distraction task, they are asked to recall as many as possible from the original list for the retrieval task (max 15). Patients’ neuropsychological scores were transformed into age-adjusted *z*-scores. Patients who underwent the AMIPB assessment had their scores compared with the respective normative data for that assessment. Patients who underwent the BMIPB assessment had their scores compared with the updated normative data from BMIPB-II. Each patient was recalled for neuropsychological evaluations at three timepoints; preoperative (AMIPB/BMIPB form 1), 3- (AMIPB/BMIPB form 2) and 12- (AMIPB/BMIPB form 1) month postoperatively.

Two outcome metrics were used to quantify the relationship of white matter on neuropsychology outcome: (i) change in neuropsychology scores between preoperative to 3 and 12 months; (ii) the reliable change index (RCI). To calculate the RCI, we used the residuals of a robust linear regression. The change in neuropsychology scores between preoperative and at 3- and 12-months postoperative was regressed against age at surgery, resection volume and preoperative neuropsychology scores. These variables have all been shown to influence changes in neuropsychology scores.^25^ An MM-estimate robust regression was utilized to mitigate the effect of outliers. The regression residuals were discretized into binary groups based on whether they were below the lower limit of an 80% confidence interval (−1, unexpected decline) or not (0, predicted change). This confidence interval was chosen based on the previous work.^26^ This metric represents clinically significant change.

## MRI data acquisition and preprocessing

Between 1995 and 2003, patients (*n* = 8) were scanned on a 1.5-T GE Signa Horizon Echospeed MR scanner (GE Medical Systems, Milwaukee, WI, USA); T1-weighted IR-SPGR volumetric acquisition (TR/TE/TI 17.4/4.2/450; 0.94 × 0.94 × 1.5 mm).

Between 2004 and 2013, patients (*n* = 89) were scanned on a 3T GE Signa Excite HDx. Single-shell dMRI data were acquired using a cardiac-triggered single-shot spin-echo planar imaging sequence: 1.875 × 1.875 × 2.4 mm resolution, gradient directions: 6 and 52 at *b*-values: 0 and 1200 s/mm^2^, δ/Δ/TE = 21/29/73 ms, and a 3D T1-weighted FSPGR sequence at 0.94 × 0.94 × 1.1 mm was acquired.^27^

Between 2014 and –2019, patients (*n* = 49) were scanned on a 3T GE Discovery MR750. Multi-shell dMRI data were acquired using 2 mm isotropic resolution, gradient directions: 11, 8, 32 and 64 at *b*-values: 0, 300, 700 and 2500 s/mm^2^; ∂/Δ=21.5/35.9 ms, TE/TR = 74.1/7600 ms. A 3D T1-weighted MPRAGE sequence at 1 × 1 × 1 mm resolution was acquired.^28^

### Diffusion preprocessing

dMRI data were denoised,^29^ Gibbs-unringed,^30^ corrected for signal drift^31^ and distortion corrected using a synthesized b0 for diffusion distortion correction (Synb0-DisCo)^32^ with FSL’s topup.^33^ Eddy currents and movement artefacts were corrected,^34^ rotating the b-vectors.^35^ Additionally, bias-field correction was performed in MRtrix3 using ANTs.^36,37^ Response functions for cerebrospinal fluid, white and grey matter were estimated using Single-Shell 3-Tissue^38^ and Multi-Shell 3-Tissue^39^ constrained spherical deconvolution in MRtrix3.

### Resection masks

For patients with a pre- and postoperative 3D T1 MRI, the preoperative scan was registered to the postoperative scan, and resection masks were manually delineated in MRtrix3 by comparing the two images, as previously reported.^40^ For patients who had only a postoperative 3D T1, this was registered to a T1 MNI152 1 mm template and missing tissue was manually delineated. Resection mask volume was normalized to the whole brain volume.

## Tractography reconstruction

**Figure 2:**
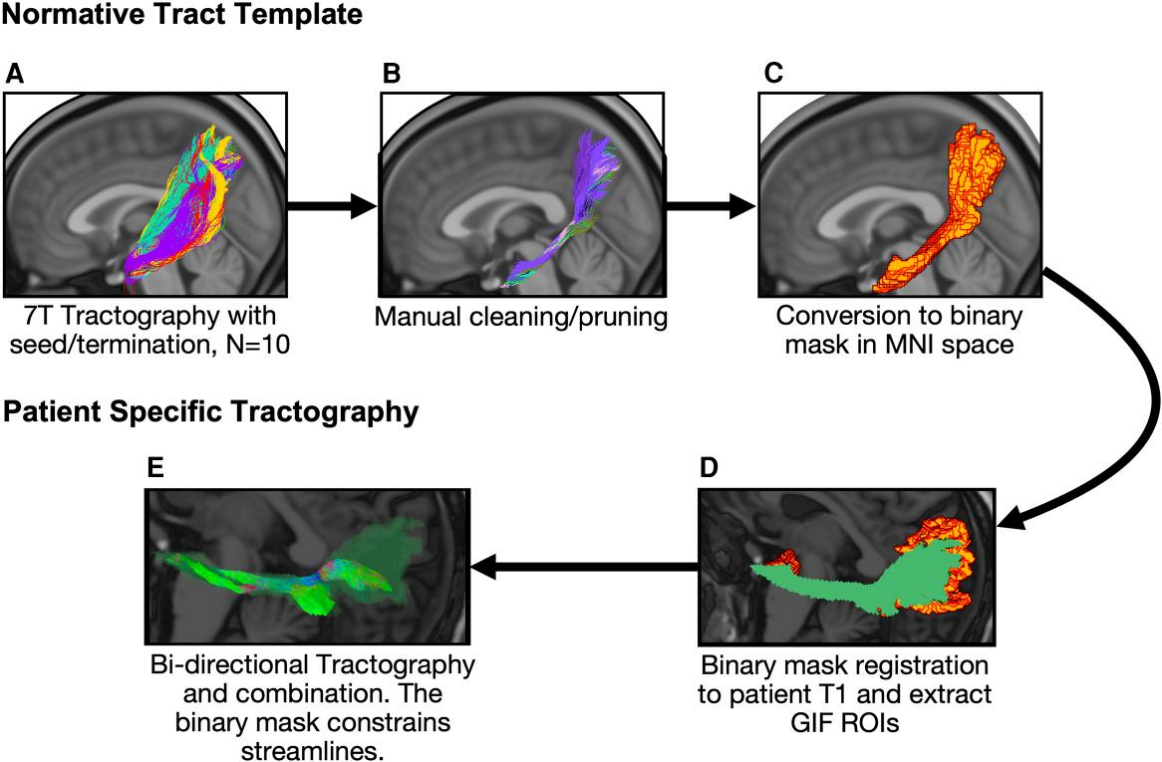
**Overview of the tractography methodology to delineate the ventral cingulum (same method was applied to the fornix)**. (**A**) Tractography was performed on 7T healthy control human connectome project dMRI data between seed/termination ROI. Seed and termination ROI were extracted by running GIF on the healthy subject’s T1 image aligned to native dMRI space. Colours represent different patients’ streamlines. (**B**) These were then manually cleaned using exclusion ROI. (**C**). Streamlines were then combined, converted to a binary mask and registered to MNI152 brain template. (**D**) The binary mask was registered to each individual patient’s T1 which was aligned in native dMRI space. GIF was used to extract the same ROI as step **A**. (**E**) Bi-directional tractography was performed using both ROI as seed and termination points. The healthy control binary mask was used to constrain streamlines, so streamlines could not leave this mask.

The fornix and ventral cingulum were reconstructed with Anatomically Targeted—Automated Tractography, an in-house algorithm modified based off the previous work.^40^ This modification incorporates anatomical priors from the human connectome project 7T dataset^41^ (Fig. 2). More information regarding tractography reconstruction is in the Supplementary material. All reconstruction methods have been made open access via https://github.com/lbinding/AT-AT.

### Cortical connections

For the ventral cingulum, the regions of interests (ROIs) included the hippocampus, parahippocampus, posterior cingulate gyrus and precuneus.^19^ For the fornix, the ROIs included the hippocampus, mammillary bodies, anterior thalamic nuclei, septal nuclei and hypothalamus.^18^

### 7T white matter anatomical priors

The Geodesic Information Flow (GIF) parcellation^42^ was used to extract cortical regions which were used as seed/termination points for tractography. Anatomically constrained tractography^43^ using hybrid surface and volume segmentation in MRtrix3^44^ was performed using second-order integration over fibre orientation distribution probabilistic fibre tracking algorithm^45^ selecting a maximum of 5000 streamlines from 300 million seeds. Tractography was performed twice, switching the seed and termination cortical ROI and merged. Fibre bundles were then converted to probabilistic maps, thresholded at a value of 0.01 and used as an exclusion criterion to remove spurious streamlines. Fibre bundles were inspected to ensure accurate reconstruction and manual exclusion masks were used to remove spurious streamlines. The cleaned tractograms were then transformed to MNI space, converted to a binary mask and dilated by 1 mm to account for co-registration errors.

### Tractography in TLE

To reconstruct the fibre bundles of interest, masks reconstructed on the MNI human connectome project 7T data were transformed to patient-native space using EasyReg.^46^ The same cortical regions and tractography parameters that were used to create the anatomical priors were used to reconstruct the fibre bundles; the 7T anatomical priors were used as an inclusive mask. Any streamlines exiting the mask were discarded. Each fibre bundle was manually inspected to ensure accuracy. This was performed on preoperative and postoperative dMRI data. Preoperative FA and mean diffusivity (MD) were extracted and the median was taken across the entire fibre bundle to reduce influence of outliers.

### Atlas-based tractograms

To perform atlas-based disconnectome analysis, we created a TLE-specific tractography atlas. In the cohort who had preoperative dMRI, the resulting fibre bundle reconstructions were converted to binary masks and transformed to MNI using EasyReg.^46^ They were combined and rescaled from 0 to 1, where 1 represents an overlap of all patients’ masks and 0 represents no overlap. This was used for atlas-based disconnectome analysis.

### Statistical analysis

#### Neuropsychology

Neuropsychology changes from preoperative to 3- and 12-month postoperative were assessed using a longitudinal linear mixed effect model. Covariates of time, age, age of onset and resection volume were included. Cases with a missing timepoint were included in the analysis. Variables that were significantly related to the change in scores were included in RCI construction.

#### Atlas-based disconnection

In the atlas-based analysis (Tractotron, part of the BCBtoolkit http://www.bcblab.com), the intersection between an individual patient’s resection mask and a percentage fibre bundle map of a TLE-based fibre bundle atlas was measured. This provides a probability of a resected voxel intersecting with a fibre bundle. This probability ranges from 0%, if resected voxels do not intersect with the fibre bundle reconstruction, to 100% if resected voxels intersect with all reconstructions of the fibre bundle. The probability provided by Tractotron reflects the probability of a resected voxel overlapping with a voxel of high probability to contain a specific fibre bundle. The value is independent of the number of resected voxels intersecting the fibre bundle, thus one resected voxel intersecting with 50% of the fibre bundle is sufficient for the fibre bundle to be classified as having a 50% probability of being disconnected.^47^

Each resection mask was registered to MNI space using easyReg.^46^ Probabilities were binarized using a threshold of 50% probability.^47^ We utilized a generalized mixed effect model to assess if tract disconnection (1) versus preservation (0) was related to RCI outcome. As the RCI accounts for covariates, we included covariates related to tract disconnection such as type of resection and resection volume.

#### Preoperative white matter integrity

We evaluated the relationship of preoperative fornix and ventral cingulum white matter integrity (FA and MD) with preoperative verbal encoding and retrieval scores. Tract integrity was scaled between 0 and 1. A Welch two-sample *t*-test was used to assess differences in scanner and microstructural metrics; significant metrics were corrected with Neurocombat. We utilized a linear regression while regressing out the age of epilepsy onset, age at surgery and resection volume.

#### Estimated resection from preoperative tractography

A direct evaluation of patient-specific fibre bundle transections was performed. This analysis measured the proportion of fibre bundle transection with changes in memory *z*-score, to investigate if there was a linear relationship between the extent of bundle transection and change in verbal memory. We calculated the percentage transection of fibre bundles by using preoperative tractography and resection masks. Resection masks were used as exclusion masks, removing any streamlines that traversed the mask to create a proxy for bundles generated from postoperative tractography. We calculated the percentage of streamlines cut for each bundle based on the comparison of the number of streamlines present in pre- and estimated postoperative tractography.

We checked to see if there was a difference between scanners and percentage of fibre bundle resection using a Welch two-sample *t*-test. If significantly different, these were corrected with NeuroCombat. We utilized a linear mixed effect model to assess if there was a linear relationship with percentage of fibre bundle resection and change in neuropsychology scores from preoperative to 3- and 12-month postoperatively. We regressed out the age of epilepsy onset, age at surgery, resection volume and preoperative neuropsychology scores and treated each subject as a random effect. To determine whether the inclusion of the tract measures provided a statistically significant improvement in model fit, we performed a likelihood ratio test by comparing the nested confound-only model to the confound-plus-tract models. We report a Chi-squared assessment between the confound-only model and confound-plus-tract models.

#### Postoperative tractography

To reinforce our findings, we reconstructed the postoperative tracts on postoperative dMRI data looked at the remaining volume to see if there was a direct relationship between remaining volume and change in memory *z*-score. Postoperative tract volume was normalized against intracranial volume and rescaled between 0 and 1. We utilized a linear mixed effect model with confounds included in the model, similar to the preoperative resection analysis.

#### Seizure outcome

International League Against Epilepsy (ILAE) outcome at 1 year was binarized into complete seizure freedom (ILAE = 1) and non-seizure freedom (ILAE > 1). A generalized mixed effect model (atlas-based disconnectome analysis) and a Welch two-sample *t*-test (preoperative tractography transection, postoperative tractography volumetric analysis) were used to investigate relationships between tract resection and seizure outcome.

## Results

For clarity and readability, this paper reports condensed statistical information where possible. Full statistical information for all analyses is in the Supplementary material.

### Descriptive statistics

**Figure 3:**
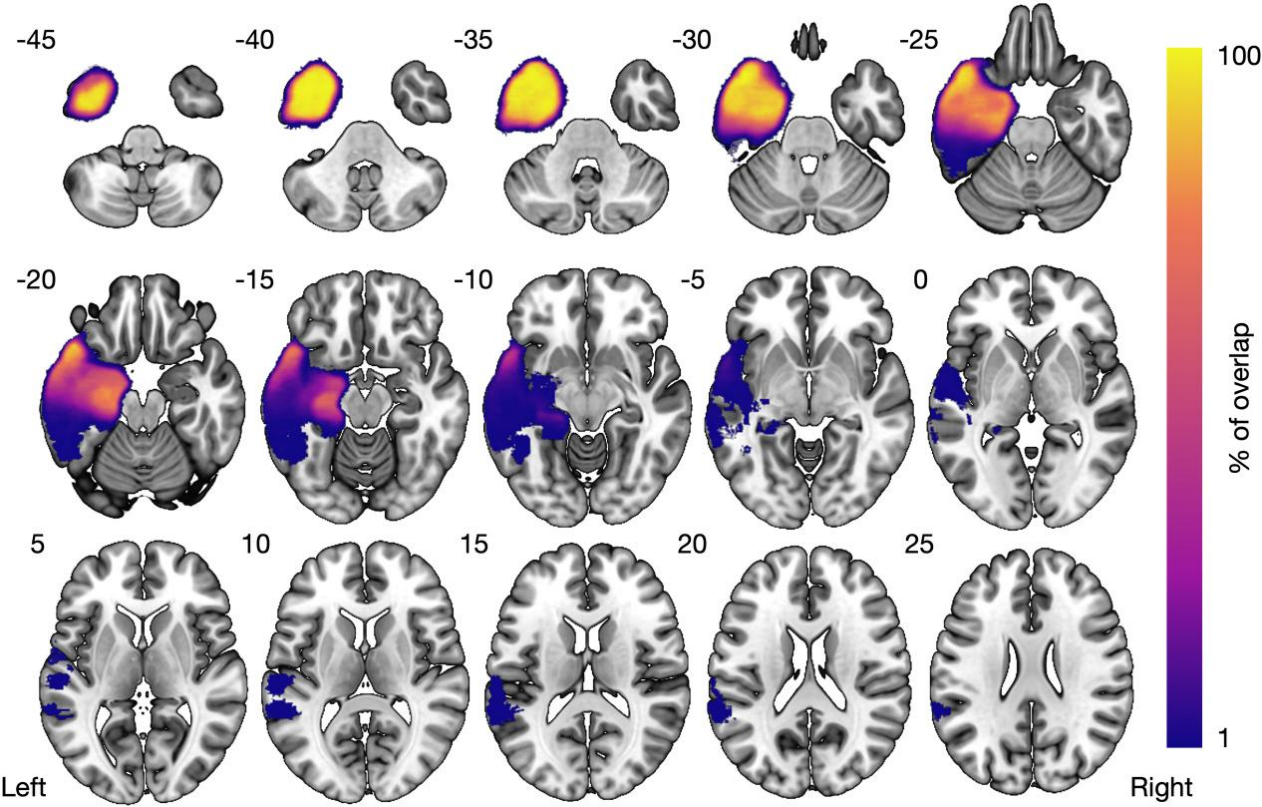
**Overlap of all resection cavities in left TLE**. All resection cavities were registered to MNI space with a colour bar showing voxel overlapping between patients.

Of the 146 patients who underwent TLE surgery, 128 had anterior temporal lobe resection and 19 had more limited temporal resections. The histopathology of specimens were hippocampal sclerosis (114), Gliosis (8), Cavernoma (9), Dysembryoplastic Neuroepithelial Tumour (6) and others (9). This study includes complete data for age and age of onset for all subjects. The following neuropsychology assessments and timepoints had missing data: verbal encoding preoperative (2), 3-month (6) and 12-month (14) postoperative; verbal retrieval preoperative (3), 3-month (4) and 12-month (15) postoperative. Preoperative tractography successfully reconstructed the fornix in 63 of 71 subjects (89%) and the ventral cingulum in 69 of 71 subjects (97%). An overlap of all resection masks can be seen in Fig. 3. Demographic details are reported in Table 1.

**Table 1:** Baseline demographic data of this cohort

	Values
Age of epilepsy onset, in years	13.00 (14.75)
Age at surgery, in years	36.00 (18.75)
Gender, F/M	78/68
Pathology (with HS), in %	78.08%
Resection type (ATLR), in %	87.67%
Proportion of brain resection	0.02 (0.01)

Values are given in median (interquartile range) unless stated otherwise. ATLR, anterior temporal lobe resection; HS, hippocampal sclerosis; F, female; M, male.

### Neuropsychology

For verbal encoding, change from preoperative scores, assessed as *z*-scores (Table 2), patient scores at 3 months were significantly worse than at 12 months (Estimate = 0.280, *P* = 0.001). Greater age at time of surgery was significantly related to greater postoperative decline, across both 3- and 12-months (Estimate = −0.025, *P* = 0.003). Resection volume and age at onset of epilepsy were not significantly related to change in scores. For RCI outcome, patients who declined had an average score change of −2.36 (STD = 0.639) at 3 months and −1.97 (STD = 0.72) at 12 months.

**Table 2:** The change in raw and *z*-score on verbal encoding and retrieval following left temporal lobe resection

Neuropsychology assessment	Timepoint (months)	Changes from preop raw score	Change from preop *z*-scores
Verbal encoding	*3*	−7.78 (10.90)	−0.89 (1.23)
	*12*	−5.24 (10.35)	−0.57 (1.2)
*Verbal retrieval*	*3*	−2.67 (3.35)	−0.85 (1.4)
	*12*	−1.39 (3.19)	−0.53 (1.28)

Values are given as the mean (standard deviation).

For verbal retrieval, change from preoperative scores (Table 2), patient scores at 3 months were significantly worse than at 12 months (Estimate = 0.344, *P* < 0.001). Greater age at time of surgery was significantly related to greater postoperative decline, across both 3- and 12-months (Estimate = −0.018, *P* = 0.046). There was a significant effect of resection volume, with greater tissue resection relating to greater postoperative decline (Estimate = −0.261, *P* = 0.014). Age at onset of epilepsy was not significantly related to change in scores. For RCI outcome, patients with decline had an average score change of −2.52 (STD = 1.22) at 3 months and −2.12 (STD = 0.66) at 12 months.

### Atlas-based disconnection

Atlas-based disconnection analysis (total cohort size: 146) was used to investigate the ventral cingulum and fornix. A generalized mixed effect model was used to account for covariates and relate tract disconnection to RCI outcome. Additional statistical information, including non-significant findings, is reported in the Supplementary material.

A total of 112 subjects (61%) had disconnection of the fornix and 120 subjects (65%) had disconnection of the ventral cingulum. Disconnection of both the ventral cingulum and the fornix was observed in 108 subjects (58%).

Patients with the ventral cingulum classified as disconnected had an increased chance of having RCI decline across both 3- and 12-month timepoints [Estimate = 1.449; X(1) = 5.833, *P* = 0.016] for verbal encoding. Patients who had, compared with patients who did not have, the ventral cingulum disconnected had an increased occurrence of RCI decline (disconnected: 22.87% versus preserved: 6.67%). The fornix was also significantly related to RCI outcome, with disconnection of the fornix being related to an increased chance of having RCI decline across timepoints [Estimate = 2.049; X(1) = 13.846, *P* < 0.001]. Patients who had, compared with patients who did not have, the fornix disconnected had an increased occurrence of RCI decline (disconnected: 24.64% versus preserved: 4.92%).

Neither the fornix nor ventral cingulum disconnection was related to verbal retrieval outcome.

Disconnection of the ventral cingulum was related to an increased chance of having an ILAE 1 seizure outcome at 12 months [disconnected: 62.50% versus preserved: 38.46%; Estimate = 1.042; X(1) = 5.334, *P* = 0.021]. Disconnection of the fornix was not related to seizure outcome.

### Preoperative white matter integrity

A linear regression was used to assess whether there was a linear relationship between fibre bundle white matter integrity and the change in neuropsychology scores (total cohort size: 71). The Chi-squared assessment between the confound-only model and confound-plus-tract models are reported. There was a significant difference in ventral cingulum [T(53.932)=−6.063, *P* < 0.001] and fornix [T(34.656)=−11.277, *P* < 0.001] FA and the ventral cingulum MD [T(49.797) = 15.363, *P* < 0.001] between scanners which was corrected with NeuroCombat. Greater age was significantly related to lower fornix (*P* < 0.001) and ventral cingulum (*P* = 0.049) FA and greater fornix MD (*P*  *<* 0.001). Additional statistical information, including non-significant findings, is reported in the Supplementary material.

We found no significant relationship between microstructure of the fornix and ventral cingulum to preoperative verbal encoding or retrieval scores.

### Preoperative tractography transection analysis

A linear mixed effect model was used to assess whether there was a linear relationship between extent of fibre bundle resection and change in neuropsychology scores (total cohort size: 71). The Chi-squared assessment between the confound-only model and confound-plus-tract models are reported. There was a significant difference in tract resection across scanners for the fornix [T(60.953) = 2.040, *P* = 0.046] which was corrected with NeuroCombat. Additional statistical information, including non-significant findings, is reported in the Supplementary material.

**Figure 4:**
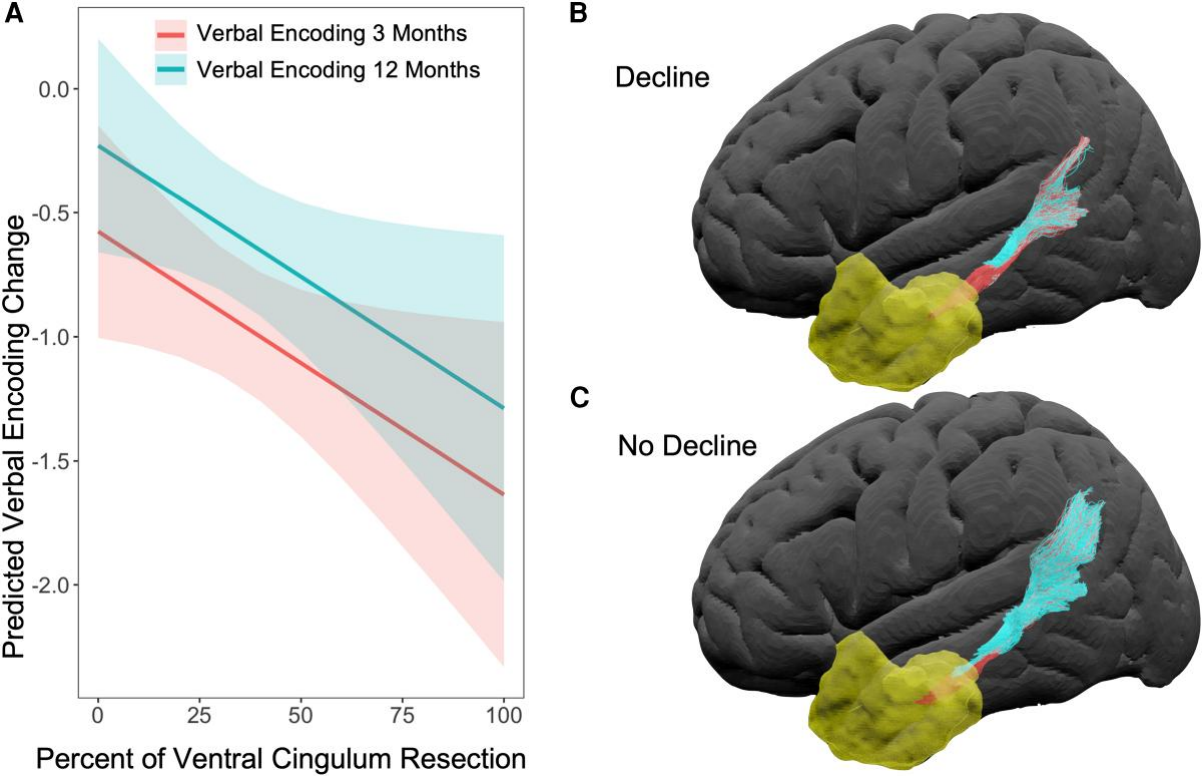
**Visualization of the ventral cingulum correlation (A) and exemplar cases (B and C)**. (**A**) Scatter plot illustrating the predicted relationship between ventral cingulum resection extent and verbal encoding change, as estimated from the linear mixed effect model (Chi-squared comparison against a null model: *P* = 0.029). Lines represent model-predicted values for each timepoint, holding age, baseline verbal encoding score, seizure type and resection mask volume constant. Shaded ribbons indicate 95% confidence intervals. Visualization of transections shown in **B** and **C** represent a patient who did (**B**) and did not (**C**) have ventral cingulum resection and verbal encoding decline. The yellow mask represents the resection mask with the intact (blue) and transected (red) fibres visualized.

For verbal encoding (Fig. 4), greater resection of the ventral cingulum was associated with significantly poorer performance across 3- and 12-month timepoints [Estimate = −0.478; X(1) = 4.741, *P* = 0.029]. The fornix was non-significant.

For verbal retrieval, greater resection of the fornix was significantly associated with poorer performance across 3- and 12-month timepoints [Estimate = −0.346; X(1) = 4.844, *P* = 0.028]. The ventral cingulum was non-significant.

There was no significant difference between seizure outcome and fornix or ventral cingulum resection.

### Postoperative tractography volumetric analysis

We used a linear mixed effect model to assess whether there was a linear relationship between remaining postoperative tract volume and change in neuropsychology (total cohort size: 47). The Chi-squared assessment between the confound-only model and confound-plus-tract models are reported. There was a significant difference in postoperative fornix volume across scanners [T(35.649) = 2.962, *P* = 0.005], this was corrected via NeuroCombat. Additional statistical information, including non-significant findings, is reported in the Supplementary material.

For verbal encoding, a greater remaining volume of the ventral cingulum [Estimate = 0.336; X(1) = 4.615, *P* = 0.032] and fornix [Estimate = 0.382; X(1) = 6.225, *P* = 0.013] was associated with better performance across 3- and 12-month timepoints.

The postoperative volume of the fornix and ventral cingulum was not significantly related to verbal retrieval or seizure outcome.

## Discussion

Memory impairments are common after temporal lobe resection for TLE and have been historically attributed to damage to the hippocampal formation. By using complementary voxel-based and tractography disconnectome analyses, our data suggest a broader temporal network involved in verbal memory encoding with deficits caused by tract transection and postoperative improvement associated with its preservation. We demonstrate that greater resection of the ventral cingulum is associated with worse postoperative verbal encoding outcomes. Paradoxically, the atlas-based analysis revealed that ventral cingulum disconnection was associated with a higher likelihood of achieving an ILAE 1 seizure outcome, despite the established link between ILAE 1 outcomes and better cognitive function, and our finding that ventral cingulum resection correlated with worse memory.

Resection involving the fornix was also associated with poorer verbal encoding outcomes in both the disconnection and postoperative volume analyses. Interestingly, the extent of fornix resection did not correlate with changes in verbal encoding, but with verbal retrieval impairments. These results suggest a critical role of the fornix and ventral cingulum in verbal memory and could impact upon how temporal lobe resections are performed.

### Direct and indirect hippocampo-thalamic pathways supporting verbal memory

Traditional models of memory are centred on the hippocampus, which is thought to link previous experiences in sequential patterns (encoding) and their retrieval.^48,49^ Our investigation does not negate the role of the hippocampus in verbal memory, but argues that larger, distributed and potentially parallel hippocampal-diencephalic networks^50^ are implicated in verbal memory loss and potentially support memory recovery.

A role for the fornix in verbal memory is established as it connects memory-associated regions such as the hippocampus, the anterior thalamic nucleus and the mammillary bodies. Lesioning the fornix causes amnesia in mice, macaques and humans.^13,14^ Our results are in line with this evidence, suggesting that fornix damage can cause deficits, and sparing this white matter pathway may support postoperative memory improvement in epilepsy patients. This is consistent with evidence from deep brain stimulation of this pathway suggesting that the fornix not only evokes detailed autobiographical memories,^51^ but can enhance memory recovery in patients with Alzheimer’s diseases.^52^

Our results also highlight the role for the ventral cingulum linking the posterior cingulate cortex and precuneus to the hippocampus and parahippocampal gyrus. The cingulum has been linked to emotion and memory since Papez,^53^ although evidence for memory deficits after its disconnection has been conflicting.^19^ It has been proposed to be the primary indirect pathway of verbal memory.^15^ Our data highlight its importance for verbal encoding and extend previous functional studies that attributed encoding of information, but not retrieval, to the left parahippocampus.^54^ The functional activation could be the result of the transference of information from the posterior cingulate cortex and precuneus. Spatial and episodic memory has been linked to the same circuit deficits after parahippocampal lesions impacting both memory functions.^48^ Spatial and episodic memory within the ventral cingulum may share computations that support cognitive maps^55^ within the verbal domain to enact encoding and retrieval of symbolic content.

We found no significant relationship between the integrity of the ventral cingulum or fornix and preoperative memory scores. This finding appears to contradict previous research suggesting that these tracts serve as direct and indirect routes for memory function, a relationship widely observed in healthy brains.^50^ However, due to the chronicity of TLE, the memory network may become less reliant on these specific tracts or interconnected regions due to ongoing seizure activity and associated functional reorganization. This hypothesis is supported by numerous fMRI studies that demonstrate reduced functional activity in the ipsilateral hippocampus and parahippocampal gyrus in TLE patients compared with healthy controls.^56,57^

Our findings suggest that while these tracts may not be critical for preoperative memory performance, their preservation is crucial for postoperative memory function. This could imply that these tracts become essential for verbal encoding and retrieval in the reorganized memory network following surgery. This is supported by recent functional connectivity research suggesting that the posterior parahippocampal gyrus’s integration into the memory network is vital for long-term (10 years+) recovery after anterior temporal lobe resection.^58,59^ Future research should aim to confirm this by conducting long-term follow-ups beyond 1 year to see if patients who had the ventral cingulum or fornix resected have worse long-term memory recovery.

By highlighting specific white matter pathways supporting episodic memory in the temporal lobe, our results shed light on the structural connections that underlie the circuit of Papez. While cortical and subcortical stations have been proposed, the white matter anatomy enabling this network have been less clear.^13,53,60,61^ Our results suggest that the fornix and the ventral cingulum have a key role. Direct connections between the hippocampus and the thalamus, and indirect connections via the neocortex, are consistent with cognitive models of memory function which posit a long-term memory component being stored in the neocortex outside the hippocampus.^49,62,63^ The ventral cingulum connects the parahippocampal to the retrosplenial cortex which may support computations within the precuneus and parietal cortex. Our analysis suggests a wider extratemporal network. A role for the prefrontal, orbitofrontal and anterior cingulate cortices, with frontal direct (anterior thalamic radiation) and indirect connections (dorsal cingulum) cannot be excluded.

The analysis of inferred tract transection found a significant association between damage to the fornix and changes in verbal retrieval, but this finding was not replicated in other analyses.

Deficits in verbal retrieval were not found to be related to damage to the fibres of the ventral cingulum or the fornix. This finding is consistent with the concept that retrieval operates as a network function^64^ and suggests that disruptions in information flow may provide additional insights into the structural network underpinning retrieval.

### Surgical relevance

Our results have implications for epilepsy surgery. Since Penfield and Baldwin,^7^ resection of the medial temporal lobe including the amygdala and hippocampus has been linked to seizure freedom. Historically, a complete resection of the hippocampus has been advocated since ictal activity was reported along its whole extent^65^ and higher rates of seizure freedom were expected for complete hippocampectomy.^66^ More recent evidence has challenged this, as quantitative analysis of hippocampal resection,^67,68^ and sparing the posterior hippocampus has not been associated with worse seizure outcome.^67^ Our results suggest that transecting the fornix may not contribute to seizure outcome while damaging it is related to memory decline.

Our atlas-based disconnection analysis demonstrated that disconnection of the ventral cingulum was associated with better seizure control. This result was not replicated in our other analyses. This finding suggests that areas that on a group level have 50% tract reconstruction overlap may contribute to seizure outcome by being part of an epileptic network. Future analysis should disentangle if white or grey matter around this tract is contributing to seizure outcome or if the ventral cingulum needs to be disconnected for seizure freedom.

A standard anterior temporal lobe resection removes the temporal pole, amygdala and anterior hippocampus and parahippocampal gyrus, resulting in disconnection of the fornix, ventral cingulum, inferior longitudinal fasciculus and often the inferior fronto-occipital fasciculus.

We previously discussed modifying the surgical approach to preserve picture naming function by avoiding the inferior fronto-occipital fasciculus and posterior parahippocampal, fusiform and inferior temporal gyri.^40^ Here, we suggest maximal avoidance of the fornix and ventral cingulum. We speculate that this could be achieved by reducing the posterior extent of hippocampal and parahippocampal resection, as these posterior regions may not impact seizure outcome but cause memory decline, in a similar fashion of how surgery has been redesigned to avoid damaging the optic radiation.^27^ Further exploration of the components of the ventral cingulum is needed to clarify whether there can be a separation between parts to be spared to preserve verbal memory and parts that need transection to give seizure control.

A caveat, however, is that long-term seizure freedom may be a consequence of transecting critical tracts, as reported in frontal lobe resections.^69^ Fornix projections are glutamatergic and connect other regions involved in temporal lobe epileptogenic networks, such as the anterior thalamic nuclei. Work is underway to assess the impact of tract disconnection on long-term seizure outcome.

### Limitations

Our work has limitations. First, resections were confined to the temporal lobe. While this may help to identify temporal regions involved in memory and seizure outcome, we have not investigated other areas that may impact upon the memory network. Second, there is variability in reconstructing small tract components with tractography (e.g. the columns of the fornix). Differences in temporal fornix extensions could be the result of anatomical differences, atrophy^70^ or incomplete bundle reconstruction. This may explain why our preoperative tractography analysis was not concordant with the rest of the analysis showing the fornix as a contributor to verbal encoding decline. We tried to overcome this using a normative white matter database created from subjects with high-resolution 7T dMRI. Future research could address these limitations by incorporating orientational priors^71^ as well as anatomical priors. Third, neuroplasticity after surgery is difficult to predict^72^ and different lesions (i.e. focal cortical dysplasias when compared with gliomas) or disease burden (i.e. frequency of seizures and duration of epilepsy) may impact recovery differently. To better understand fibre bundle transection and postoperative plasticity, task-based or resting-state fMRI could be utilized to jointly explore functional and structural change within the memory network.

## Conclusion

Episodic memory impairments are frequent in TLE and following temporal lobe epilepsy surgery. In agreement with classical^53^ and contemporary^61^ distributed models of episodic memory, our data point to parallel hippocampal-diencephalic networks supporting this in the temporal lobe and shed light on the structural connections that underlie the circuit of Papez. Our results argue in favour of verbal memory being a network function and have implications for clinical practice. We show that disconnection of the fornix and ventral cingulum may be associated with memory decline and sparing these fibres could result in less memory decline. Redesigning surgical procedures to maximize seizure freedom^68^ while minimizing cognitive deficit^40^ could make surgical intervention a more attractive option.

## Supplementary Material

fcag033_Supplementary_Data

## Data Availability

The method and code for this analysis will be made openly available via: https://github.com/lbinding/MemoryPaper. Anonymized data will be made available upon request from any qualified investigator.
