# Supplementary material for: White matter resection and verbal memory deficits after temporal lobe epilepsy surgery
Source: Brain Commun. 2026 Feb 2;8(2):fcag033. doi: 10.1093/braincomms/fcag033 (PMC12977961; doi:10.1093/braincomms/fcag033)
Supplement: fcag033_Supplementary_Data [file fcag033_supplementary_data.docx]

# **Descriptive Statistics**

# **Neuropsychology**

## **Verbal Encoding**

### Linear mixed effect model

| Variable | Statistic |
| --- | --- |
| Timepoint 3-months | Estimate = -0.917, p < 0.000, 95CI = -1.103:-0.731 |
| Timepoint 12-months | Estimate = -0.623, p < 0.000, 95CI = -0.813:-0.434 |
| Age | Estimate = -0.029, p < 0.000, 95CI = -0.045:-0.014 |
| Resection Mask | Estimate = -0.343, p < 0.000, 95CI = -0.524:-0.162 |
| Onset Age | Estimate = 0.009, p = 0.292, 95CI = -0.008:0.026 |
| Random effects | Variance = 0.985, STD = 0.993 |

### Post-hoc T-test with Bonferroni Correction

|  | LTLE Preop | LTLE 3m postop | LTLE 12m postop |
| --- | --- | --- | --- |
| LTLE Preop | - | - | - |
| LTLE 3m postop | *p* < 0.001 | - | - |
| LTLE 12m postop | *p <* 0.001 | *p* = 0.160 | - |

## **Verbal Retrieval**

### Repeated Measures ANOVA (Across Three Timepoints)

| Variable | Statistic |
| --- | --- |
| Timepoint 3-months | Estimate = -0.892, p < 0.000, 95CI = -1.101:-0.683 |
| Timepoint 12-months | Estimate = -0.555, p < 0.000, 95CI = -0.770:-0.341 |
| Age | Estimate = -0.015, p = 0.100, 95CI = -0.033:0.003 |
| Resection Mask | Estimate = -0.314, p = 0.004, 95CI = -0.520:-0.108 |
| Onset Age | Estimate = 0.001, p = 0.880, 95CI = -0.017:0.020 |
| Random effects | Variance = 1.2800, STD = 1.1314 |

### Post-hoc T-test with Bonferroni Correction

|  | LTLE Preop | LTLE 3m postop | LTLE 12m postop |
| --- | --- | --- | --- |
| LTLE Preop | - | - | - |
| LTLE 3m postop | *p* < 0.001 | - | - |
| LTLE 12m postop | *p* = 0.007 | *p* = 0.115 | - |

# **Atlas-based Disconnection**

## **Verbal Encoding**

### Fornix (GLMM)

| Variable | Statistic |
| --- | --- |
| Timepoint | Estimate = -0.028, p = 0.932, 95CI = -0.683:0.626 |
| Resection Mask | Estimate = -0.394, p = 0.128, 95CI = -0.900:0.113 |
| Resection Type | Estimate = -1.945, p = 0.036, 95CI = -3.767:-0.123 |
| Onset Age | Estimate = 0.007, p = 0.671, 95CI = -0.026:0.040 |
| Fornix Disconnection | Estimate = 2.049, p = 0.003, 95CI = 0.708:3.390 |
| Random Effect (Subjects) | Variance = 0.691, Std. Dev. = 0.831 |
| Overall Model | X(1)=13.846, p < 0.001 |

### Ventral Cingulum (GLMM)

| Variable | Statistic |
| --- | --- |
| Timepoint | Estimate = -0.007, p = 0.983, 95CI = -0.654:0.640 |
| Resection Mask | Estimate = -0.342, p = 0.178, 95CI = -0.840:0.156 |
| Resection Type | Estimate = -1.618, p = 0.074, 95CI = -3.396:0.160 |
| Onset Age | Estimate = 0.002, p = 0.910, 95CI = -0.031:0.035 |
| Ventral Cingulum Disconnection | Estimate = 1.449, p = 0.033, 95CI = 0.118:2.780 |
| Random Effect (Subjects) | Variance = 0.756, Std. Dev. = 0.869 |
| Overall Model | X(1)=5.833, p = 0.016 |

## **Verbal Retrieval**

### Fornix (GLMM)

| Variable | Statistic |
| --- | --- |
| Timepoint | Estimate = -0.061, p = 0.872, 95CI = -0.802:0.681 |
| Resection Mask | Estimate = -0.131, p = 0.701, 95CI = -0.799:0.537 |
| Resection Type | Estimate = -3.141, p = 0.025, 95CI = -5.893:-0.389 |
| Onset Age | Estimate = 0.043, p = 0.072, 95CI = -0.004:0.090 |
| Fornix Disconnection | Estimate = 0.614, p = 0.328, 95CI = -0.615:1.843 |
| Random Effect (Subjects) | Variance = 3.233, Std. Dev. = 1.798 |
| Overall Model | X(1)=0.986, p = 0.321 |

### Ventral Cingulum (GLMM)

| Variable | Statistic |
| --- | --- |
| Timepoint | Estimate = -0.066, p = 0.862, 95CI = -0.811:0.679 |
| Resection Mask | Estimate = -0.122, p = 0.724, 95CI = -0.802:0.558 |
| Resection Type | Estimate = -3.178, p = 0.028, 95CI = -6.010:-0.346 |
| Onset Age | Estimate = 0.043, p = 0.080, 95CI = -0.005:0.091 |
| Ventral Cingulum Disconnection | Estimate = 0.035, p = 0.961, 95CI = -1.362:1.433  > |
| Random Effect (Subjects) | Variance = 3.414, Std. Dev. = 1.848 |
| Overall Model | X(1)=0.002, p = 0.96 |

## **Seizure Outcome**

### Fornix (GLMM)

| Variable | Statistic |
| --- | --- |
| Resection Mask | Estimate = -0.397, p = 0.091, 95CI = -0.856:0.063 |
| Resection Type | Estimate = -1.010, p = 0.154, 95CI = -2.398:0.379 |
| Age | Estimate = 0.014, p = 0.364, 95CI = -0.016:0.045 |
| Onset Age | Estimate = -0.014, p = 0.421, 95CI = -0.047:0.020 |
| Fornix Disconnection | Estimate = 0.654, p = 0.116, 95CI = -0.163:1.471 |
| Random Effect (Subjects) | Variance = 0.018, Std. Dev. = 0.135 |
| Overall Model | X(1)=2.598, p = 0.107 |

### Ventral Cingulum (GLMM)

| Variable | Statistic |
| --- | --- |
| Resection Mask | Estimate = -0.395, p = 0.095, 95CI = -0.859:0.069 |
| Resection Type | Estimate = -0.859, p = 0.227, 95CI = -2.254:0.536 |
| Age | Estimate = 0.014, p = 0.360, 95CI = -0.017:0.046 |
| Onset Age | Estimate = -0.017, p = 0.324, 95CI = -0.051:0.017 |
| Ventral Cingulum Disconnection | Estimate = 1.042, p = 0.030, 95CI = 0.099:1.986 |
| Random Effect (Subjects) | Variance = 0.024, Std. Dev. = 0.156 |
| Overall Model | X(1)=5.334, p = 0.021 |

# **Preoperative Tractography Transection Analysis**

## **Scanner Differences**

| Variable | Statistics |
| --- | --- |
| Fornix (FNX) | T(60.953)=2.040, *p* = 0.046 |
| Ventral Cingulum (vCing) | T(60.843)=0.789, *p* = 0.433 |

## **Verbal Encoding**

### Fornix

| Variable | Statistic |
| --- | --- |
| Timepoint | Estimate = 0.264, p = 0.076, 95CI = -0.012:0.565 |
| Pre-operative scores | Estimate = -0.460, p = 0.000, 95CI = -0.670:-0.250 |
| Percentage of FNX Resection | Estimate = -0.250, p = 0.131, 95CI = -0.549:0.051 |
| Age | Estimate = -0.042, p = 0.003, 95CI = -0.067:-0.017 |
| Resection Type | Estimate = -0.544, p = 0.490, 95CI = -1.988:0.906 |
| Resection Volume | Estimate = -0.295, p = 0.263, 95CI = -0.776:0.187 |
| Random Effect (Subjects) | Variance = 0.535, Std. Dev. = 0.732 |
| Overall Model | X(1)=2.675, p = 0.102 |

#### Model Without Resection Volume

| Variable | Statistic |
| --- | --- |
| Timepoint | Estimate = 0.264, p = 0.076, 95CI = -0.013:0.565 |
| Pre-operative scores | Estimate = -0.434, p = 0.000, 95CI = -0.642:-0.225 |
| Percentage of FNX Resection | Estimate = -0.320, p = 0.040, 95CI = -0.602:-0.037 |
| Age | Estimate = -0.046, p = 0.001, 95CI = -0.071:-0.022 |
| Resection Type | Estimate = 0.119, p = 0.819, 95CI = -0.854:1.093 |
| Random Effect (Subjects) | Variance = 0.538, Std. Dev. = 0.733 |
| Overall Model | X(1)=4.884, p = 0.027 |

### Ventral Cingulum

| Variable | Statistic |
| --- | --- |
| Timepoint | Estimate = 0.348, p = 0.006, 95CI = 0.110:0.588 |
| Pre-operative scores | Estimate = -0.037, p = 0.000, 95CI = -0.056:-0.019 |
| Percentage of vCing Resection | Estimate = -0.478, p = 0.000, 95CI = -0.645:-0.311 |
| Age | Estimate = -0.275, p = 0.038, 95CI = -0.522:-0.028 |
| Resection Type | Estimate = -0.181, p = 0.694, 95CI = -1.051:0.690 |
| Resection Volume | Estimate = -0.129, p = 0.415, 95CI = -0.429:0.170 |
| Random Effect (Subjects) | Variance = 0.450, Std. Dev. = 0.671 |
| Overall Model | X(1)=4.741, p = 0.029 |

## **Verbal Retrieval**

### Fornix

| Variable | Statistic |
| --- | --- |
| Timepoint | Estimate = 0.656, p = 0.001, 95CI = 0.290:1.035 |
| Pre-operative scores | Estimate = -0.289, p = 0.013, 95CI = -0.494:-0.084 |
| Percentage of FNX Resection | Estimate = -0.346, p = 0.042, 95CI = -0.652:-0.040 |
| Age | Estimate = -0.031, p = 0.040, 95CI = -0.058:-0.004 |
| Resection Type | Estimate = 1.030, p = 0.216, 95CI = -0.491:2.554 |
| Resection Volume | Estimate = 0.148, p = 0.581, 95CI = -0.345:0.643 |
| Random Effect (Subjects) | Variance = 0.480, Std. Dev. = 0.693 |
| Overall Model | X(1)=4.844, p = 0.028 |

#### Model Without Resection Volume

| Variable | Statistic |
| --- | --- |
| Timepoint | Estimate = 0.656, p = 0.001, 95CI = 0.290:1.033 |
| Pre-operative scores | Estimate = -0.305, p = 0.006, 95CI = -0.504:-0.105 |
| Percentage of FNX Resection | Estimate = -0.313, p = 0.047, 95CI = -0.601:-0.025 |
| Age | Estimate = -0.029, p = 0.045, 95CI = -0.055:-0.002 |
| Resection Type | Estimate = 0.692, p = 0.212, 95CI = -0.338:1.722 |
| Random Effect (Subjects) | Variance = 0.470, Std. Dev. = 0.686 |
| Overall Model | X(1)=4.511, p = 0.034 |

### Ventral Cingulum

| Variable | Statistic |
| --- | --- |
| Timepoint | Estimate = 0.587, p = 0.000, 95CI = 0.279:0.890 |
| Pre-operative scores | Estimate = -0.430, p = 0.000, 95CI = -0.604:-0.256 |
| Percentage of vCing Resection | Estimate = -0.198, p = 0.220, 95CI = -0.501:0.105 |
| Age | Estimate = -0.021, p = 0.094, 95CI = -0.044:0.002 |
| Resection Type | Estimate = 0.381, p = 0.503, 95CI = -0.691:1.454 |
| Resection Volume | Estimate = -0.187, p = 0.338, 95CI = -0.554:0.181 |
| Random Effect (Subjects) | Variance = 0.657, Std. Dev. = 0.810 |
| Overall Model | X(1)=1.668, p = 0.197 |

#### Model Without Resection Volume

| Variable | Statistic |
| --- | --- |
| Timepoint | Estimate = 0.585, p = 0.000, 95CI = 0.276:0.888 |
| Pre-operative scores | Estimate = -0.426, p = 0.000, 95CI = -0.601:-0.252 |
| Percentage of vCing Resection | Estimate = -0.254, p = 0.092, 95CI = -0.537:0.030 |
| Age | Estimate = -0.021, p = 0.085, 95CI = -0.045:0.002 |
| Resection Type | Estimate = 0.722, p = 0.106, 95CI = -0.120:1.564 |
| Random Effect (Subjects) | Variance = 0.470, Std. Dev. = 0.686 |
| Overall Model | X(1)=3.081, p = 0.079 |

## **Seizure Outcome**

| Variable | Statistic |
| --- | --- |
| Fornix | T(50.588)=-0.177, *p* = 0.871 |
| Ventral Cingulum | T(27.569)=-0.614, *p* = 0.545 |

# **Preoperative White Matter Integrity Analysis**

## **Scanner Differences**

| Variable | Statistics |
| --- | --- |
| Fornix (FNX) FA | T(34.656)=-11.277, *p* < 0.001 |
| Fornix (FNX) MD | T(39.823)=1.843, *p* < 0.073 |
| Ventral Cingulum (vCing) FA | T(53.932)=-6.063, *p* < 0.001 |
| Ventral Cingulum (vCing) MD | T(49.797)=15.363, *p* < 0.001 |

## **Age correlation**

| Variable | Statistics |
| --- | --- |
| Fornix (FNX) FA | Estimate = -0.042, *p* < 0.001, CI=-0.06:-0.02 |
| Fornix (FNX) MD | Estimate = 0.036, *p <* 0.001, CI=0.02:0.06 |
| Ventral Cingulum (vCing) FA | Estimate = -0.023, *p* = 0.049, CI=-0.05:0.00 |
| Ventral Cingulum (vCing) MD | Estimate = -0.009, *p* = 0.449, CI=-0.03:0.01 |

## **Verbal Encoding**

### vCing FA

| Variable | Statistic |
| --- | --- |
| vCing FA | Estimate = -0.075, p = 0.664, 95CI = -0.418:0.268 |
| Age | Estimate = -0.014, p = 0.377, 95CI = -0.044:0.017 |
| Resection Type | Estimate = 0.063, p = 0.938, 95CI = -1.554:1.679 |
| Resection Volume | Estimate = -0.316, p = 0.219, 95CI = -0.824:0.193 |
| Overall Model | X(1)=0.190, p = 0.664 |

### vCing MD

| Variable | Statistic |
| --- | --- |
| vCing MD | Estimate = 0.050, p = 0.761, 95CI = -0.276:0.375 |
| Age | Estimate = -0.012, p = 0.449, 95CI = -0.042:0.019 |
| Resection Type | Estimate = 0.132, p = 0.868, 95CI = -1.444:1.708 |
| Resection Volume | Estimate = -0.295, p = 0.235, 95CI = -0.787:0.197 |
| Overall Model | X(1)=0.093, p = 0.76 |

### Fornix FA

| Variable | Statistic |
| --- | --- |
| FNX FA | Estimate = 0.032, p = 0.860, 95CI = -0.329:0.393 |
| Age | Estimate = -0.011, p = 0.524, 95CI = -0.045:0.023 |
| Resection Type | Estimate = 0.170, p = 0.832, 95CI = -1.431:1.771 |
| Resection Volume | Estimate = -0.277, p = 0.259, 95CI = -0.763:0.209 |
| Overall Model | X(1)=0.031, p = 0.860 |

### Fornix MD

| Variable | Statistic |
| --- | --- |
| FNX MD | Estimate = 0.097, p = 0.575, 95CI = -0.248:0.443 |
| Age | Estimate = -0.018, p = 0.256, 95CI = -0.050:0.014 |
| Resection Type | Estimate = 0.168, p = 0.831, 95CI = -1.401:1.737 |
| Resection Volume | Estimate = -0.283, p = 0.244, 95CI = -0.764:0.198 |
| Overall Model | X(1)=0.318, p = 0.575 |

## **Verbal Retrieval**

### vCing FA

| Variable | Statistic |
| --- | --- |
| vCing FA | Estimate = 0.019, p = 0.926, 95CI = -0.384:0.421 |
| Age | Estimate = 0.022, p = 0.226, 95CI = -0.014:0.059 |
| Resection Type | Estimate = 0.590, p = 0.538, 95CI = -1.315:2.495 |
| Resection Volume | Estimate = -0.011, p = 0.972, 95CI = -0.617:0.596 |
| Overall Model | X(1)=0.009, p = 0.926 |

### vCing MD

| Variable | Statistic |
| --- | --- |
| vCing MD | Estimate = -0.110, p = 0.560, 95CI = -0.488:0.267 |
| Age | Estimate = 0.021, p = 0.251, 95CI = -0.015:0.056 |
| Resection Type | Estimate = 0.605, p = 0.513, 95CI = -1.235:2.445 |
| Resection Volume | Estimate = 0.014, p = 0.961, 95CI = -0.566:0.595 |
| Overall Model | X(1)=0.344, p = 0.560 |

### Fornix FA

| Variable | Statistic |
| --- | --- |
| FNX FA | Estimate = -0.170, p = 0.416, 95CI = -0.584:0.245 |
| Age | Estimate = 0.015, p = 0.467, 95CI = -0.025:0.054 |
| Resection Type | Estimate = 0.428, p = 0.647, 95CI = -1.434:2.289 |
| Resection Volume | Estimate = -0.044, p = 0.878, 95CI = -0.614:0.526 |
| Overall Model | X(1)=0.671, p = 0.416 |

### Fornix MD

| Variable | Statistic |
| --- | --- |
| FNX MD | Estimate = 0.026, p = 0.896, 95CI = -0.377:0.430 |
| Age | Estimate = 0.018, p = 0.355, 95CI = -0.020:0.056 |
| Resection Type | Estimate = 0.536, p = 0.560, 95CI = -1.296:2.368 |
| Resection Volume | Estimate = -0.031, p = 0.913, 95CI = -0.598:0.536 |
| Overall Model | X(-1)=0.017, p = 0.896 |

# **Postoperative Tractography Volumetric Analysis**

## **Scanner Differences**

| Variable | Statistics |
| --- | --- |
| Fornix (FNX) | T(35.649)=2.962, *p* = 0.005 |
| Ventral Cingulum (vCing) | T(33.914)=-0.483, *p* = 0.632 |

## **Verbal Encoding**

### Fornix

| Variable | Statistic |
| --- | --- |
| Timepoint | Estimate = 0.259, p = 0.081, 95CI = -0.017:0.557 |
| Pre-operative scores | Estimate = -0.490, p = 0.000, 95CI = -0.693:-0.285 |
| Postop FNX Volume | Estimate = 0.382, p = 0.022, 95CI = 0.085:0.678 |
| Age | Estimate = -0.042, p = 0.002, 95CI = -0.066:-0.018 |
| Resection Type | Estimate = -1.143, p = 0.149, 95CI = -2.579:0.299 |
| Resection Volume | Estimate = -0.372, p = 0.118, 95CI = -0.803:0.059 |
| Random Effect (Subjects) | Variance = 0.478, Std. Dev. = 0.691 |
| Overall Model | X(1)=6.225, p = 0.013 |

### Ventral Cingulum

| Variable | Statistic |
| --- | --- |
| Timepoint | Estimate = 0.291, p = 0.040, 95CI = 0.030:0.578 |
| Pre-operative scores | Estimate = -0.428, p = 0.001, 95CI = -0.639:-0.217 |
| Postop vCing Volume | Estimate = 0.336, p = 0.047, 95CI = 0.031:0.641 |
| Age | Estimate = -0.051, p = 0.001, 95CI = -0.076:-0.025 |
| Resection Type | Estimate = -0.899, p = 0.275, 95CI = -2.410:0.617 |
| Resection Volume | Estimate = -0.300, p = 0.243, 95CI = -0.771:0.172 |
| Random Effect (Subjects) | Variance = 0.599, Std. Dev. = 0.774 |
| Overall Model | X(1)=4.615, p = 0.032 |

## **Verbal Retrieval**

### Fornix

| Variable | Statistic |
| --- | --- |
| Timepoint | Estimate = 0.659, p = 0.001, 95CI = 0.294:1.040 |
| Pre-operative scores | Estimate = -0.302, p = 0.014, 95CI = -0.520:-0.083 |
| Postop FNX Volume | Estimate = -0.021, p = 0.910, 95CI = -0.366:0.323 |
| Age | Estimate = -0.026, p = 0.092, 95CI = -0.055:0.002 |
| Resection Type | Estimate = 0.953, p = 0.294, 95CI = -0.711:2.620 |
| Resection Volume | Estimate = -0.058, p = 0.829, 95CI = -0.553:0.438 |
| Random Effect (Subjects) | Variance = 0.543, Std. Dev. = 0.637 |
| Overall Model | X(1)=0.160, p = 0.689 |

#### Model Without Resection Volume

| Variable | Statistic |
| --- | --- |
| Timepoint | Estimate = 0.660, p = 0.001, 95CI = 0.294:1.040 |
| Pre-operative scores | Estimate = -0.296, p = 0.013, 95CI = -0.509:-0.083 |
| Percentage of FNX Resection | Estimate = -0.015, p = 0.935, 95CI = -0.355:0.325 |
| Age | Estimate = -0.027, p = 0.070, 95CI = -0.055:0.000 |
| Resection Type | Estimate = 1.089, p = 0.094, 95CI = -0.109:2.287 |
| Random Effect (Subjects) | Variance = 0.563, Std. Dev. = 0.750 |
| Overall Model | X(1)=0.010, p = 0.922 |

### Ventral Cingulum

| Variable | Statistic |
| --- | --- |
| Timepoint | Estimate = 0.583, p = 0.003, 95CI = 0.222:0.959 |
| Pre-operative scores | Estimate = -0.269, p = 0.023, 95CI = -0.483:-0.056 |
| Postop vCing Volume | Estimate = -0.004, p = 0.981, 95CI = -0.345:0.337 |
| Age | Estimate = -0.026, p = 0.087, 95CI = -0.053:0.002 |
| Resection Type | Estimate = 0.788, p = 0.380, 95CI = -0.870:2.447 |
| Resection Volume | Estimate = -0.080, p = 0.767, 95CI = -0.582:0.422 |
| Random Effect (Subjects) | Variance = 0.590, Std. Dev. = 0.768 |
| Overall Model | X(1)=0.000, p = 0.987 |

#### Model Without Resection Volume

| Variable | Statistic |
| --- | --- |
| Timepoint | Estimate = 0.584, p = 0.003, 95CI = 0.222:0.960 |
| Pre-operative scores | Estimate = -0.263, p = 0.022, 95CI = -0.473:-0.053 |
| Percentage of vCing Resection | Estimate = 0.006, p = 0.975, 95CI = -0.330:0.341 |
| Age | Estimate = -0.027, p = 0.063, 95CI = -0.053:-0.000 |
| Resection Type | Estimate = 0.977, p = 0.120, 95CI = -0.187:2.140 |
| Random Effect (Subjects) | Variance = 0.568, Std. Dev. = 0.754 |
| Overall Model | X(1)=0.002, p = 0.968 |

## **Seizure Outcome**

| Variable | Statistic |
| --- | --- |
| Fornix | T(41.702)=-1.757, *p* = 0.086 |
| Ventral Cingulum | T(42.908)=-0.027, *p* = 0.979 |
